# Socio-economic and household determinants of malaria in adults aged 45 and above: analysis of longitudinal ageing survey in India, 2017–2018

**DOI:** 10.1186/s12936-021-03840-w

**Published:** 2021-07-07

**Authors:** Indumathi Mohan, Naveen Kumar Kodali, Savitha Chellappan, Balasubramani Karuppusamy, Sujit Kumar Behera, Gopalan Natarajan, Praveen Balabaskaran Nina

**Affiliations:** 1 Department of Epidemiology and Public Health, Central University of Tamil Nadu, Tiruvarur, Tamil Nadu India; 2 ICMR-National Institute of Traditional Medicine, Belagavi, India; 3 Department of Geography, Central University of Tamil Nadu, Tiruvarur, Tamil Nadu India

**Keywords:** LASI, Socio-economic determinants of malaria, Household determinants of malaria, Schedule tribe and malaria, Unclean cooking fuel and malaria

## Abstract

**Background:**

Even though malaria cases have drastically come down in the last decade, malaria remains a serious public health concern in many parts of India. National Framework for Malaria Elimination in India (2016–2030) has been launched with the goal to eliminate malaria by 2030. Understanding the socio-economic and household determinants of malaria at the national level will greatly aid India’s malaria elimination efforts.

**Methods:**

The data from Longitudinal Ageing Survey of India (LASI) Wave 1 (2017–2018) survey comprising 70,671 respondents ≥ 45 years across all the States and Union Territories were used for the analysis. Simple and multiple logistic regressions were used to obtain the unadjusted and adjusted odds ratio respectively of the socio-economic and household variables.

**Results:**

The major socio-economic variables that increase the likelihood of malaria are caste (‘scheduled tribes’), low education levels and rural residence. The scheduled tribes have 1.8 times higher odds of malaria than the scheduled castes (AOR: 1.8; 95% CI: 1.5–2.1). Respondents with high school education (6–12 grade) (AOR: 0.7; 95% CI: 0.6–0.8) and college education (AOR: 0.5; 95% CI: 0.4–0.6) had a very low risk of malaria than those with no school years. Rural residence and occupation (agriculture and allied jobs) also increases the odds of malaria. The major housing determinants are household size (≥ 6), housing type (kutcha), use of unclean fuel, outside water source, improper sanitation (toilet facilities) and damp wall/ceiling.

**Conclusions:**

The study has identified the major socio-economic and housing factors associated with malaria in adults aged 45 and above. In addition to vector and parasite control strategies in the tribal dominated regions of India, improving literacy and housing conditions may help India’s malaria elimination efforts.

## Background

Globally, vector borne diseases account for > 17% of all the infectious diseases, and account for > 700,000 deaths annually [1]. Malaria is a major vector borne disease, and is a serious public health concern in many parts of India [2–5]. Malaria situation in India is complex with varied distribution of *Plasmodium vivax* and *Plasmodium falciparum* [6]. According to the World Health Organization (WHO), 93% of the population in India are at risk of malaria [7]. From 2000 to 2017, malaria morbidity and mortality in India have declined by 59 and 89%, respectively [8]. In India, malaria is transmitted by several *Anopheles* spp., and the geography determines the primary *Anopheles* vector; *Anopheles stephensi*, *Anopheles culicifacies*, *Anopheles dirus*, *Anopheles fluviatilis*, *Anopheles minimus* and *Anopheles sundaicus* are considered to be the primary malaria vectors in India [9].

In 2015, India has committed to elimination of malaria by 2030 at the Asia Pacific Leaders Malaria Alliance meeting in Kuala Lampur [10]. The National Vector Borne Disease Control Programme (NVBDCP) launched the National Framework for Malaria Elimination in India (2016–2030) in 2016 with two major goals: (1) Eliminating malaria throughout India by 2030 and (2) Maintaining malaria-free status in regions where malaria transmission is disrupted, and preventing re-introduction of malaria [11]. Furthermore, Malaria Elimination Research Alliance has been launched under the umbrella of Indian Council of Medical Research (ICMR) to “*identify, articulate, prioritize and respond to the research needs of the country to eliminate malaria from India by 2030*” [8].

India’s malaria control strategies focuses on effective vector control using indoor residual spraying, long-lasting insecticidal nets, chemical insecticides, bacterial pesticides and larvivorous fish [12]. Chemotherapy using artemisinin-based combination therapy for *P. falciparum* and chloroquine/primaquine for *P. vivax* are the major treatment strategies under the national drug policy [13].

Malaria transmission is influenced by several factors, including socio-economic and demographic characteristics of the study area [14]. In addition, housing factors have also been shown to play an important role in malaria transmission [15, 16]. In India, studies on socio-economic and household determinants of malaria are very limited, and are focused on selected districts [14, 17–19]. Type of house, toilet facility and water-source were the major housing risk factors [17], while, social groups, family size [14], monthly income [18] were some of the key socio-economic determinants reported in these Indian studies.

A pan-India study on the socio-economic and household determinants of malaria may give important insights on the major risk factors, and aid in country’s malaria control and elimination efforts. Longitudinal Ageing survey of India-1 (LASI-1) carried out across India from April 2017 to December 2018 provides important insight into various health parameters of elderly individuals (≥ 45 years), including the socio-economic and housing conditions of self-reported cases of malaria in the past two years before the survey. This LASI data set from 70,671 individuals was used to analyse the key socio-economic and housing determinants of malaria, and the results are detailed.

## Methods

### Data and participants

Data from the LASI Wave 1 (2017–2018) survey carried out by the International Institute for Population Sciences (IIPS) in Mumbai across 28 states (except Sikkim) and seven Union Territories (UT) were used for the analysis. A multi-stage cluster sampling was used to collect data on many social, economic and health indicators. The LASI Wave-1 surveyed 70,671 respondents ≥ 45 years and their spouses (even if they are < 45). There were 28,754 (41 %) aged 45–54 years, 28,579 (40.4 %) aged 55–69 years, 13,338 (19 %) aged ≥ 70 years, and 40,877 (58 %) females. The LASI has employed Computer-Assisted Personal Interview techniques to record the responses of survey participants.

### Study variables

#### Outcome variable

The outcome of interest is malaria, and was based on the following question: 1. In the past 2 years, have you had malaria? The options were: (1) Yes, and (2) No. The response was coded as a binary variable (No—0: absence of malaria; Yes—1: presence of malaria).

#### Socio-economic status and demographic variables

The socio-economic status (SES) and demographic variables used for this analysis are age-group (45–54 years, 55–69 years and ≥ 70 years), sex (male or female), place of residence (rural or urban), income category based on Monthly Per capita Consumption Expenditure (MPCE) (poorest, poor, middle, richer and richest), educational level (0 school years, 1–5 school years, 6–12 school years and college), work (not working, agricultural and allied, self-employed and wage/salary worker), and caste (SC, ST, OBC and forward caste). The terms ‘SC’ (Scheduled Castes) and ‘ST’ (Scheduled Tribes) are officially used in the government documents to identify the socially weaker sections and tribes in the country. The major class of the country is OBC (Other Backward Classes), and comprises 40–50 % of the population.

#### Household conditions

Household variables used are household size (1–5/≥ 6 members), type of house (pucca/kutcha), location of water source (own dwelling, yard/plot or outside dwelling), toilet type (improved: flushed to piped sewer system/septic tank/pit latrine/twin pit/composting toilet; unimproved sanitation: open defecation), cooking fuel (clean fuel: LPG, biogas and electricity; unclean fuel: kerosene, charcoal, coal, crop residue, wood/shrub and dung cake) and damp wall or ceiling (yes/no).

### Statistical analysis

A frequency distribution table was prepared for all the variables used in this study. Prevalence of malaria with each of the SES (age group, sex, residence area, education level, MPCE quintiles, caste, and work) and household variables (household size, type of house, location of water source, toilet type, cooking fuel, and damp wall or ceiling) were reported. Simple logistic regression was used to obtain the unadjusted odds ratio (UOR), and those independent variables found significant (P < 0.05) were included in the multiple logistic regression to arrive at the Adjusted Odds Ratio (AOR) of SES and household variables in association with malaria. All the independent variables were significant at the alpha value of 5 % except for the variable ‘sex’ in the univariable logistic regression analysis. The variable ‘sex’ was used in the multivariable logistic regression despite not found significant in the univariable analysis because ‘sex’ is an important variable to adjust for in the analysis. Sampling weights calculated by LASI were applied during the analysis to obtain accurate estimates. All analyses were performed using STATA MP statistical software version 16.

### Spatial analysis

The malaria prevalence (%) data was added as attribute in State and UT boundaries of India using ArcGIS software. The histogram of the malaria prevalence data was analysed, and was used to determine the four class intervals for mapping; the national average of malaria period prevalence is 7.91 %. The rounded off class interval 0.1–5.0 represents below national average States/UT, 5.1–10.0 represents national average, 10.1–15.0 denotes above national average, and 15.1–25.0 shows twice the national average. The choropleth techniques were used for mapping, and darker colors denote higher values.

## Results

### Prevalence of malaria in adults ≥ 45 years

**Fig. 1:**
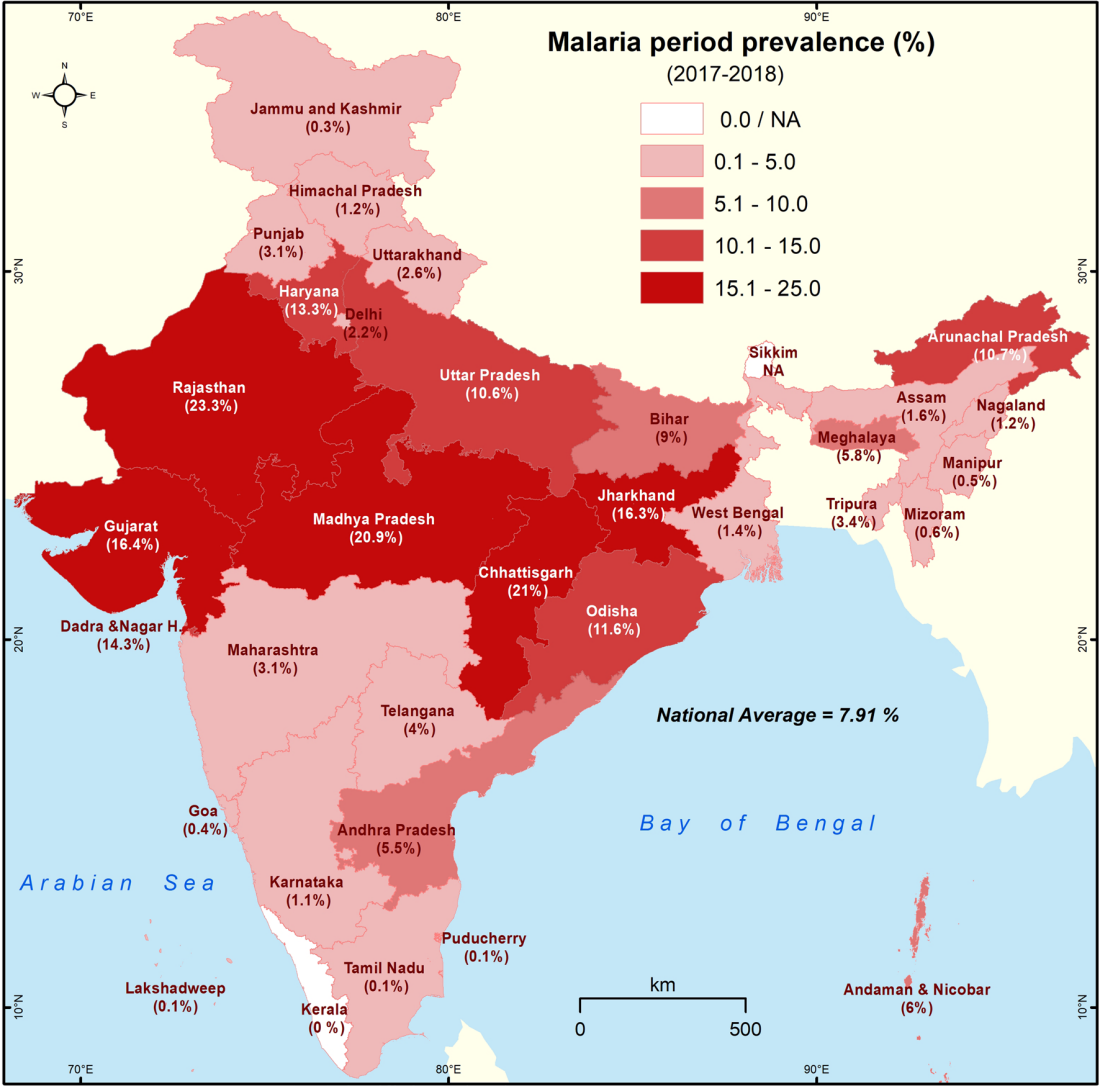
Self-reported prevalence of malaria in different States and Union Territories of India (LASI-1 2017–2018). The intervals represent malaria prevalence. The darker the shade, higher is the prevalence

The frequency distribution of all the study variables is shown in Table 1. The prevalence of malaria during the period 2017–2018 in adults ≥ 45 years is 7.9 %. The self-reported period prevalence of malaria across all the States and UT of India during the survey is shown in Fig. 1. The period prevalence of malaria is high in Central and Western India when compared to the South, North and Eastern regions. The states with very high (> 15 %) prevalence include Rajasthan (23.3 %), Chhattisgarh (21 %), Madhya Pradesh (20.9 %), Gujarat (16.4 %) and Jharkhand (16.3 %). The above national average (10–15 %) prevalence is reported in the States/UT surrounding Central India─Dadra and Nagar Haveli (14.3 %), Haryana (13.3 %), Odisha (11.6 %), Uttar Pradesh (10.6 %) and Bihar (9 %). The States/UT that exceeded 5 % prevalence in the South are Andhra Pradesh (5.5 %) and Andaman & Nicobar Islands (6 %), and in the North-East (NE), it is Arunachal Pradesh (10.7 %) and Meghalaya (5.8 %). The northern-most States/UT (Jammu & Kashmir, Himachal Pradesh, Punjab and Uttarakhand) show very low (0–5 %) prevalence of malaria.

**Table 1:** Distribution of socio-demographic and household variables of adults ≥ 45 years in India, LASI-1 (2017–2018)

Variables	n	%
Malaria	5589	7.9
Age group (44–54 years)	28,754	40.7
55–69 years	28,579	40.4
≥ 70 years	13,338	18.9
Sex (female)	40,877	57.8
Male	29,795	42.2
Residence (rural)	48,921	69.2
Urban	21,750	30.8
MPCE quintile (poorest)	14,790	20.9
Poorer	15,023	21.3
Middle	14,268	20.2
Richer	13,781	19.5
Richest	12,809	18.1
Education (0 school years)	35,081	49.6
1–5 school years	12,334	17.5
6–12 school years	18,883	26.7
College	4372	6.2
Caste (SC)	13,612	20.0
ST	6033	8.9
OBC	31,530	46.3
Forward	16,924	24.9
Main job (not woking)	35,590	50.1
Agricultural and allied	19,961	28.1
Self-employed	6072	8.55
Wage/salary worker	9406	13.2
Household-size (1–5 members)	44,815	63.0
≥ 6 members	26,319	37.0
Type of house (pucca/semi pucca)	58,912	83.3
Kutcha	11,834	16.7
Water source (own dwelling/plot/yard)	46,231	68.7
Outside dwelling	21,056	31.3
Toilet facility (improved sanitation)	51,652	73.4
Unimproved	18,766	26.7
Cooking fuel (clean)	36,858	52.3
Unclean	33,559	47.7
Damp wall/ceiling (no)	55,684	79.1
Yes	14,748	20.9

The prevalence of malaria associated with SES and household variables in adults ≥ 45 is shown in Table 2. Malaria prevalence is similar in all age groups, and is slightly higher in males (8.1 %) than in females (7.8 %). Malaria is higher in the rural (9.6 %) than in urban (4 %) respondents. The prevalence of malaria is highest in the ST population (15 %) when compared to the other social groups. Malaria prevalence reduced with increasing levels of education (9.7 % in illiterates vs. 3 % in the college educated) and income of households (9.2 % in the poorest vs. 6.9 % in the richest). Malaria prevalence is highest in those working in agriculture and allied jobs (10.5 %), and is lowest in wage/salaried workers (5.2 %). Malaria is highest in households with ≥ six members (9.5 %), and in those living in kutcha houses (12 %). The prevalence of malaria is more in adults with no source of drinking water inside the house (10.6 %) than those who have a water-source within their residence (6.9 %). Malaria is more in households (11.7 %) with poor sanitation (unimproved toilet facility) compared to households (5 %) that have improved sanitation facilities. Malaria in households using unclean fuel is 11.2 %, and in households with damp wall/ceiling, it is 9.6 %.

**Table 2:** Prevalence of malaria by socio-demographic and household variables in adults ≥ 45 years in India, LASI-1 (2017–2018)

Variables	n	%	Total
Age group (44–54 years)	2107	7.3	28,757
55–69 years	2415	8.4	28,591
≥ 70 years	1067	8.0	13,340
Sex (female)	3170	7.8	40,884
Male	2419	8.1	29,803
Residence (rural)	4717	9.6	48,931
Urban	873	4.0	21,756
MPCE quintile (poorest)	1366	9.2	14,794
Poorer	1293	8.6	15,026
Middle	1072	7.5	14,267
Richer	972	7.1	13,791
Richest	885	6.9	12,810
Education (0 school years)	3400	9.7	35,087
1–5 school years	1049	8.5	12,336
6–12 school years	1010	5.3	18,890
College	130	3.0	4373
Caste (SC)	1118	8.2	13,612
ST	904	15.0	6035
OBC	2387	7.6	31,542
Forward	1086	6.4	16,927
Main job (not working)	2691	7.6	35,369
Agricultural and allied	2076	10.5	19,837
Self-employed	331	5.5	6043
Wage/salary worker	485	5.2	9333
Household-size (1–5 members)	3117	7.0	44,571
≥ 6 Members	2472	9.5	26,116
Type of house (pucca/semi pucca)	4166	7.1	58,552
Kutcha	1402	11.9	11,751
Water source (own dwelling/plot/yard)	3236	7.0	45969
Outside dwelling	2214	10.6	20897
Toilet facility (Improved sanitation)	3374	6.5	51663
Unimproved	2204	11.7	18771
Cooking fuel (clean)	1828	5.0	36875
Unclean	3751	11.2	33557
Damp wall/ceiling (No)	4160	7.5	55684
Yes	1419	9.6	14748

### Association between socio-economic and housing conditions with malaria in adults ≥ 45 years

The SES and household variables analysed by simple and multiple logistic regressions are shown in Table 3. Rural residence, richest, illiterates, less educated, ST population, working in agriculture and allied jobs, not working, household size with ≥ six members, no water-source within a dwelling, unimproved toilet facility, using unclean fuel for cooking, and damp wall/ceiling are associated with an increased risk for malaria.

**Table 3:** Odds ratios of malaria in adults ≥ 45 years, LASI-1 (2017–2018)

Variables	Malaria		
	UOR	AOR	95 % CI
Age group (44–54 years)	1		
55–69 years	1.2**	1.0	0.9–1.1
70 + years	1.1	0.9	0.8–1.1
Sex (female)	1		
Male	1.1	1.2**	1.0–1.3
Residence (rural)	1		
Urban	0.4**	0.7**	0.6–0.8
MPCE quintile (poorest)	1		
Poorer	0.9	1.1	1.0–1.2
Middle	0.8**	1.0	0.9–1.2
Richer	0.7**	1.1	0.9–1.2
Richest	0.7**	1.2**	1.1–1.5
Education (0 school years)	1		
1–5 school years	0.8*	1.0	0.9–1.1
6–12 school years	0.5**	0.7**	0.6–0.8
College/university	0.3**	0.5**	0.4–0.7
Caste (SC)	1		
ST	1.9**	1.7**	1.5–1.9
OBC	0.9	1.1	1.0–1.2
Forward	0.8**	1.0	0.9–1.2
Main Job (wage/salary worker)	1		
Agricultural and allied	2.1**	1.3*	1.1–1.5
Self-employed	1.1	1	0.8–1.3
Not working	1.5**	1.3**	1.1–1.5
Household size (1–5 members)	1		
≥ 6 members	1.4**	1.3**	1.2–1.4
Type of house (pucca/semi pucca)	1		
Kutcha	1.8**	1.1**	1.0–1.3
Water source (own dwelling/yard/plot)	1		
Outside dwelling	1.6**	1.1**	1.0–1.2
Toilet facility (improved sanitation)	1		
Unimproved	1.9**	1.2**	1.1–1.3
Cooking fuel (clean)	1		
Unclean	2.4**	1.5**	1.4–1.7
Damp wall/ceiling (no)	1	1	
Yes	1.3**	1.2**	1.1–1.3

*UOR* unadjusted odds ratio, *AOR* adjusted odds ratio

*P<0.05 ** P<0.01

Residing in an urban area reduced the risk of malaria (AOR: 0.7; 95 % CI: 0.6–0.8). Males have slightly higher odds for malaria than females (AOR: 1.1; 95 % CI: 1.0-1.3). Richest are 1.2 times more at risk for malaria than the poorest (AOR: 1.3; 95 % CI: 1.1–1.5). Respondents who did high school education (6–12 grade) (AOR: 0.7; 95 % CI: 0.6–0.8) and college education (AOR: 0.5; 95 % CI: 0.4–0.6) have a very low risk of malaria than those with no school education. The ST have 1.8 times higher odds of malaria than the SC (AOR: 1.8; 95 % CI: 1.5–2.1). Malaria odds are higher for those working in agriculture and allied jobs, and in those who are ‘not working’ (AOR: 1.3; 95 % CI: 1.1–1.5) when compared to wage/salaried workers. Households with more than five members have a higher likelihood for malaria (AOR: 1.5; 95 % CI: 1.3–2.7). Respondents in kutcha houses have more odds for malaria (AOR: 1.1; 95 % CI: 1.1–1.3) than those in pucca houses. Households with water-source not in the dwelling (AOR: 1.1; 95 % CI: 1.1–1.4), and unimproved toilet facility (AOR: 1.2; 95 % CI: 1.1–1.3) have more odds for malaria. Households using unclean fuel for cooking have 1.5 times higher odds (AOR: 1.5; 95 % CI: 1.4–1.7) when compared to households that are using LPG/electricity/biogas for cooking. Also, households with damp wall/ceiling have 1.2 times higher odds of malaria (AOR: 1.2 95 % CI 1.1–1.3) than those with no damp wall/ceiling.

## Discussion

According to LASI (2017-18), the States/UT with over 10 % prevalence include Rajasthan, Chhattisgarh, Madhya Pradesh, Gujarat, Jharkhand, Dadra and Nagar Haveli, Haryana, Odisha, Uttar Pradesh and Arunachal Pradesh. Among these, Odisha, Chhattisgarh, Jharkhand and Madhya Pradesh are highly endemic for malaria. From July 2019, High Burden to High Impact strategy of WHO has been initiated in Jharkhand, Chhattisgarh, Madhya Pradesh and West Bengal [20, 21]. Considering the known endemicity of malaria in different States, the overall trends of malaria prevalence are on expected lines. However, there are surprising high and low prevalence data of malaria in specific States. Two striking observations stand-out in this survey: (1) Rajasthan at 23.3 % ranks number 1 in the self-reported cases and (2) The NE State of Mizoram is among the lowest with 0.5 %. Even though, malaria (esp. *P. vivax*) is prevalent in Rajasthan, it is not considered to be among the top 5 malaria endemic states in India [22, 23]. On the other hand, Mizoram is considered to be one of the highly malaria endemic States in India [24, 25]. One explanation for the unexpected numbers could be the study sites where the survey was undertaken. For example, in Mizoram, the district of Aizawl reported 57 malaria cases in 2018, while the malaria-endemic districts of Lawngtlai, Lunglei and Mamit reported 2222, 1092 and 772, respectively. Therefore, if the survey was carried out at Aizawl, the capital of Mizoram, the self-reported malaria cases will be lower. Another possibility could be the adults in Mizoram may be asymptomatic due to various types of adaptive or acquired immunity [26]. In sub-Saharan Africa, many adults who harbour the parasites rarely show clinical symptoms [26]. At Mamit, the average annual parasite index (API) from 2010 to 2018 was 34.4 (34 cases / 1000), one of the highest in the country [24]. During 2014 to 2015, there was a big spike in malaria cases in Mizoram, and in Mamit district, nearly 50 % of the total population (8766 cases out of 17,731) were affected in 2015 [24].

Despite the significant strides India has made in decreasing malaria mortality and morbidity in the last two decades (from 2000 to 2019, malaria cases and deaths have declined by 71.8 and 73.9 %, respectively) [27], malaria remains a serious public health issue in several parts of the country. The NVBDCP has developed a comprehensive strategic plan to achieve malaria-free India by 2030 [28]. For devising effective malaria control and elimination strategies, understanding the socio-economic and household variables that affect malaria transmission is imperative. Analysis indicates rural residence, occupation (agricultural and allied), education levels (illiterates and primary), caste (ST), household size (≥ 6), sanitation (poor toilet facility), unclean cooking fuel, water-source not in dwelling, damp wall/ceiling are the major socio-economic and household risk factors that affect malaria transmission.

Not surprisingly, ST population are at a higher malaria risk. Jharkhand, Chhattisgarh, Madhya Pradesh, Odisha and malaria-endemic NE States (Tripura, Meghalaya, Mizoram and Arunachal Pradesh) have a high ST population. The geographical terrain that includes many forested areas, poor accessibility, frequent natural hazards, perennial *P. falciparum* transmission, very efficient anthropophillic vectors, and socio-cultural practices greatly hinder malaria control efforts in many rural tribal areas of India [6, 29]. The orthodox health beliefs of tribal population have restrained them from accessing health services despite them being highly vulnerable to various health hazards, including malaria [29, 30]. In addition, the dense forest cover and high rainfall in the tribal belts are conducive for mosquito breeding, and malaria transmission [29, 30]. Many of the tribal pockets where malaria is endemic are characterized by poor housing conditions. Residents in kutcha houses have higher odds of malaria; kutcha houses may have holes and gaps that allow easy entry of mosquitoes. This is in line with earlier Indian studies where kutcha houses/walls made of dung and earth have shown to be a risk factor for malaria [14, 17]. Positive association between mosquito bites/day and bamboo houses has been reported in Assam, India [18].

Literacy has a negative association with malaria; illiterates and those with just primary education have higher risk of malaria. This was expected as literacy gives a better understanding of infectious diseases and the protective measures required. However, earlier studies [14, 18] in India did not find an association between education and malaria risk. In Yadav et al. [18], the sample size was just 71 households, while in Sharma et al. [14], only no schooling, primary and secondary grades were included, and college education was not included in the education characteristics. ST who are at higher risk of malaria have lower literacy rate (59 %), when compared to the national average of 73 % [31].

Malaria risk is higher in those who carry out agricultural and allied activities when compared to respondents who are self-employed or get wage/salary. Agricultural activities require significant time to be spent outdoors, and these individuals are at higher risk of mosquito bites. A recent study from Mandla district in Madhya Pradesh shows households having own farmlands to have a significant association with malaria [17].

Size of the household (≥ 6 members) contributed a significant risk to malaria prevalence. Family size/number of people in the house/number of people per room is an important risk factor for malaria [14, 17, 32–35], as crowding attracts more mosquitoes due to strong olfactory signals [36]. As observed in studies carried out in India [14, 17], Ethiopia [37], Indonesia [38], and sub-Saharan Africa [39], access to outside water-source is a major malaria risk factor, as dependence on outside source for water, especially in dusk and dawn increases the chances of mosquito bite. Furthermore, households using tube-wells as outside water-source have risk of malaria [17], as tube-wells are suggested to have more stagnant water around them due to improperly maintained drainage facilities [40]. Poor toilet facility (sanitation) is another important household risk factor of malaria, and is in-line with earlier studies carried out in India [17] and elsewhere [37–39, 41]. Use of unclean cooking fuel is also a major risk factor (odds increase by 1.5 times) for malaria. In addition, damp wall/ceiling is also associated with increased malaria risk; damp walls favour indoor resting of mosquitoes [42]. The three household determinants: outside water-source, improper toilet facility and unclean cooking fuel increases the likelihood of mosquito bites outside the house. Increased time required for outdoor cooking using unclean cooking fuel could be a reason for its higher odds. Free clean cooking fuel (liquid petroleum gas connection) has been given to > 80 million Indian households through the Pradhan Mantri Ujjwala Yojana (PMUY) scheme [43]. Through this scheme, it is expected that 80 % of the households will have clean cooking fuel by 2019 [44]. In addition to improving the standard of living, the PMUY scheme may also help in malaria control and elimination efforts.

Malaria is considered to be a disease of the poor [45–47], and several studies have shown significant association between poverty and malaria [48, 49]. There are also studies that have shown no significant association between malaria and SES of the household [17, 50–52]. Interestingly, even though prevalence of malaria is higher in poorest, after adjusting the other socio-economic variables, richest were found to have slightly higher risk of malaria than the other economic categories. Urban malaria is predominantly caused by *P. vivax*, and as this is a pan-India study, a higher proportion of respondents positive for *P. vivax* could have been from urban cities, and are likely to be socio-economically forward. For example, Uttar Pradesh, the most populous State in India has predominantly *P. vivax* [23]. Furthermore, richest, especially in urban cities may get tested promptly, and report accurately. However, the socio-economic-housing risk factors like rural residence, caste (ST), education levels, housing conditions, sanitation, unclean cooking fuel, improper water source and damp wall/ceiling strongly suggest poverty to be a risk factor for *P. falciparum* malaria, especially in the tribal dominated States of Jharkhand, Chhattisgarh, Madhya Pradesh, Odisha, Mizoram, Tripura and Meghalaya.

The major limitation of the study is that it is limited to adults ≥ 45 years old. Malaria affects all age groups, and this study captures only a particular age group. Furthermore, as malaria prevalence is self-reported, the accuracy cannot be verified.

## Conclusions

Overall, the study gives important insights on socio-economic and housing determinants of malaria. In parallel to parasite and vector control strategies, improving the socio-economic and living conditions, especially in malaria dominated tribal pockets may assist the malaria elimination efforts.

## Data Availability

The datasets supporting the conclusions of this article are included within the article.

## Abbreviations

LASI: Longitudinal Ageing Survey of India
IIPS: International Institute for Population Sciences
ICMR: Indian Council for Medical Research
AOR: Adjusted odds ratio
UOR: Unadjusted odds ratio
NVBDCP: National Vector Borne Disease Control Programme
NE: North-East
ST: Scheduled Tribes
SC: Scheduled Castes
PMUY: Pradhan Mantri Ujjwala Yojana
SES: Socio-economic status
UT: Union Territories
